# Systolic Strain Abnormalities to Predict Hospital Readmission in Patients With Heart Failure and Normal Ejection Fraction

**DOI:** 10.4021/cr104w

**Published:** 2011-11-20

**Authors:** Steven M. Borer, Aravind Kokkirala, David M. O'Sullivan, David I. Silverman

**Affiliations:** a Henry Low Heart Center, Hartford Hospital, Hartford, CT, USA

**Keywords:** Heart failure, Echocardiography, Strain, Outcomes

## Abstract

**Background:**

Despite intensive investigation, the pathogenesis of heart failure with normal ejection fraction (HFNEF) remains unclear. We hypothesized that subtle abnormalities of systolic function might play a role, and that abnormal systolic strain and strain rate would provide a marker for adverse outcomes.

**Methods:**

Patients of new CHF and left ventricular ejection fraction > 50% were included. Exclusion criteria were recent myocardial infarction, severe valvular heart disease, severe left ventricular hypertrophy (septum >1.8 cm), or a technically insufficient echocardiogram. Average peak systolic strain and strain rate were measured using an off-line grey scale imaging technique. Systolic strain and strain rate for readmitted patients were compared with those who remained readmission-free.

**Results:**

One hundred consecutive patients with a 1st admission for HFNEF from January 1, 2004 through December 31, 2007, inclusive, were analyzed. Fifty two patients were readmitted with a primary diagnosis of heart failure. Systolic strain and strain rates were reduced in both study groups compared to controls. However, systolic strain did not differ significantly between the two groups (-11.7% for those readmitted compared with -12.9% for those free from readmission, P = 0.198) and systolic strain rates also were similar (-1.05 s^-1^ versus -1.09 s^-1^, P = 0.545). E/e’ was significantly higher in readmitted patients compared with those who remained free from readmission (14.5 versus 11.0, P = 0.013). E/e’ (OR 1.189, 95% CI 1.026-1.378; P = 0.021) was found to be an independent predictor for HFNEF readmission.

**Conclusions:**

Among patients with new onset HFNEF, SS and SR rates are reduced compared with patients free of HFNEF, but do not predict hospital readmission. Elevated E/e’ is a predictor of readmission in these patients.

## Introduction

Despite four decades of impressive progress, congestive heart failure remains the most frequent reason for admission to hospital within the United States, and its impact continues to increase as the US population ages. While both medical and mechanical treatments have produced measurable improvement in survival and quality of life, the social and economic costs of caring for heart failure patients continues to accelerate [1]. Mortality rates exceed 30% for patients admitted to hospital for a 1st episode of heart failure, and approach 15% annually for heart failure patients overall [2, 3]. Nearly half of patients with heart failure demonstrate normal systolic function [4], and readmission rates for patients with heart failure with normal ejection fraction (HFNEF) are indistinguishable from those who demonstrate systolic dysfunction [5]. In ominous contrast to systolic heart failure, no therapeutic agent or strategy to date has been shown to alter mortality in HFNEF patients.

Although clinical classification of patients with HFNEF would appear to be straightforward [6], agreement as to both etiology and pathogenesis has generated ongoing debate, as the prevailing wisdom that diastolic dysfunction explains the disorder has been repeatedly challenged [7-9]. The difficulty of facile, accurate, non-invasive assessment of diastolic function adds to the controversy. In particular, the challenge of separating out load-independent abnormalities of intrinsic relaxation from the loading conditions (left atrial and left ventricular end-diastolic pressure) that accompany them has limited progress in explanation of pathophysiology and rational classification of HFNEF patients [10]. Other lines of investigation suggest that subtle abnormalities of systolic function play a role despite the presence of a normal ejection fraction [11-13].

Routine identification and assessment of systolic dysfunction in patients with HFNEF might add valuable prognostic information in the ongoing treatment of these patients, and strain imaging may provide an optimal tool for this task. Strain imaging has been shown to be a highly sensitive tool for the identification of subtle abnormalities of systolic function in other settings, most notably during exercise-induced ischemia [14]. In HFNEF patients systolic strain values are reduced at rest and fail to rise appropriately with exercise [15]. To date, the potential prognostic value of systolic strain and strain rate has not been defined in patients with HFNEF. We hypothesized that systolic strain and strain rate would be reduced in the presence of HFNEF, and that patients with abnormal systolic strain and strain rate would suffer from a higher incidence of hospital readmission and longer length of stay.

## Methods

### Patients and data collection

One hundred consecutive patients discharged from Hartford Hospital with a primary diagnosis of new onset congestive heart failure from January 1, 2004 through December 31, 2007, inclusive, were analyzed. Inclusion criteria were age ≥ 18 and an LVEF > 50% demonstrated by 2-dimensional echocardiography performed during the index hospitalization. Exclusion criteria were a prior hospital admission for CHF, an echocardiogram technically insufficient to allow for accurate strain analysis (determined by visual assessment of the study investigators), severe mitral or aortic stenosis (valve area < 1 cm^2^) or regurgitation, severe left ventricular hypertrophy (septal wall thickness > 1.8 cm), myocardial infarction within 6 weeks of admission, or previous mitral or aortic valve replacement. Baseline demographics (age, gender, presence of hypertension, diabetes mellitus, and index hospitalization length of stay), echocardiographic parameters (LVEF, LV end-systolic and end-diastolic dimensions, septal and posterior wall thickness, left atrial size, peak mitral E wave and A wave velocities, E/A ratio, septal e’, E/e’, and peak systolic velocity (Sa), and plasma brain natriuretic peptide (BNP) levels were recorded. Ninety percent of BNP levels were obtained within the 1st 24 hours of admission. Biplane LVEF was measured by modified Simpson’s rule using an offline digital analysis system (Agfa Heartlab, Greenville, SC). The study was approval by the hospital’s Institutional Review Board.

Once included into the study database, each patient’s echocardiogram was analyzed by the study investigators. Apical 4 chamber views were imported into the Axius™ Velocity Vector Imaging (VVI) (Siemens Medical Solutions, Mountain View CA), an offline analysis program that can perform velocity, strain, and strain rate analysis using a validated grey scale imaging technique [16, 17]. The endocardial border of the left ventricle during end-systole was traced in the apical 4 chamber view using 7 to 11 tracking points. Manual readjustments were made as necessary to ensure that endocardial borders were accurately traced. All tracings were performed by a single member of the study team (SMB). A random sample of 10% of cases were retraced and validated by a second study member (DIS), with < 20% inter-observer variability. Peak velocity, myocardial strain, and strain rate measurements were made in the six available segments of the apical 4 chamber view, and then averaged as a total apical 4 chamber score. This process was repeated for a total of three measurements per patient, and the three scores were averaged and recorded.

A control group was composed of patients admitted without heart failure who had an echocardiogram for an alternative indication (most commonly cardiac source of embolus) performed during the incident hospitalization. Potential controls with an LVEF < 50% (by Simpson’s rule), prior history of congestive heart failure, septal wall thickness > 1.4 cm), and moderate or severe valvular stenosis or regurgitation were excluded. Baseline demographics and echocardiographic parameters were recorded, as performed with the HFNEF patients.

### Outcomes and statistics

The primary endpoint was readmission for CHF, defined as readmission to hospital within the study period with a diagnosis related group (DRG) for CHF listed in the top three admission diagnoses. Secondary endpoints were all-cause readmission, and readmission for CHF or all cause within one year. Accepting a 20% difference in strain and strain rate between patients who were readmitted and those who were not, a sample size of 100 (50 in each group) was shown to afford 80% power to detect an effect size of 0.60 using a two group t-test with a 0.050 two-sided significance level.

Univariate analyses were performed to look for preliminary differences in all variables between groups. Normally distributed, continuous variables (e.g., age) were evaluated with t-tests, and categorical variables (e.g., gender) were evaluated with chi-square tests. Binary logistic regression modeling was used to evaluate possible predictors of readmission. All statistical analyses were performed with SPSS version 14.0 (SPSS Inc., Chicago IL 2006) at an alpha level of 0.05, such that results yielding P < 0.05 were deemed statistically significant.

## Results

Fifty two of the 100 patients analyzed in this study were readmitted with a primary diagnosis of heart failure; 48 patients remained free from readmission (Table 1). The two groups were similar with regard to age, gender, presence of hypertension, diabetes, BNP level, LVEF, echocardiographic dimensions, peak mitral inflow velocities and tissue Doppler imaging velocities (Table 1). In a subset of patients who received tissue Doppler assessment (available after 2005, n = 57), E/e’ was significantly higher in readmitted patients compared with those who remained free from readmission (14.5 versus 11.0, P = 0.013).

**Table 1:** Baseline Demographics and Echocardiographic Parameters for Hospital Readmission for CHF

variable	Not readmitted for CHF (n = 48)	Readmitted for CHF (n = 52)	P value
Age (years)	76.17	77.55	0.602
Index length of stay (days)	4.88	5.35	0.481
Males (n, %)	13 (0.27)	21 (0.40)	0.206
Females (n, %)	35 (0.73)	31 (0.60)	0.206
Hypertension (n, %)	22 (0.45)	23 (0.44)	0.872
Diabetes (n, %)	16 (0.51)	26 (0.50)	0.154
BNP pg/mL	882	956	0.788
LVEF (%)	67	65	0.120
LVIDd (cm)	4.3	4.5	0.182
LVIDs (cm)	2.8	3.0	0.208
Septal thickness (cm)	1.2	1.3	0.084
Post wall thickness (cm)	1.12	1.20	0.360
LA dimension (cm)	4.2	4.1	0.704
Peak mitral E (cm/s)	97	93	0.520
E/a	1.6	1.4	0.236
E’ (cm/s)	8.5	7.7	0.263
E/e’	11.0	14.5	0.013*
Sa (cm/s)	7.7	7.5	0.619
Systolic strain (%)	-12.91	-11.74	0.198
Systolic strain rate (s^-1^)	-1.09	-1.05	0.545

^*^values are statistically significant at P < 0.05.

**Figure 1:**
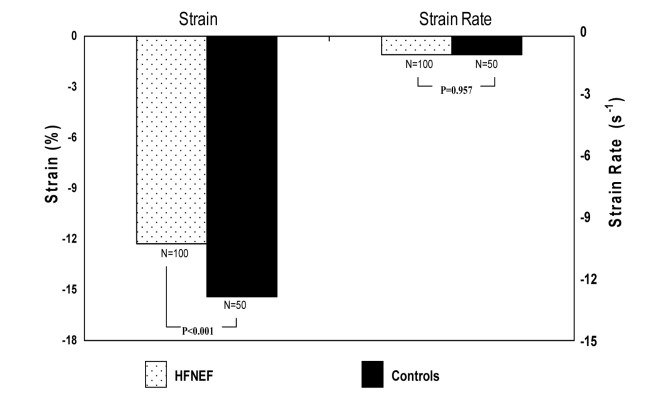
Systolic strain and strain rate values for HFNEF Patients and Controls.

**Figure 2:**
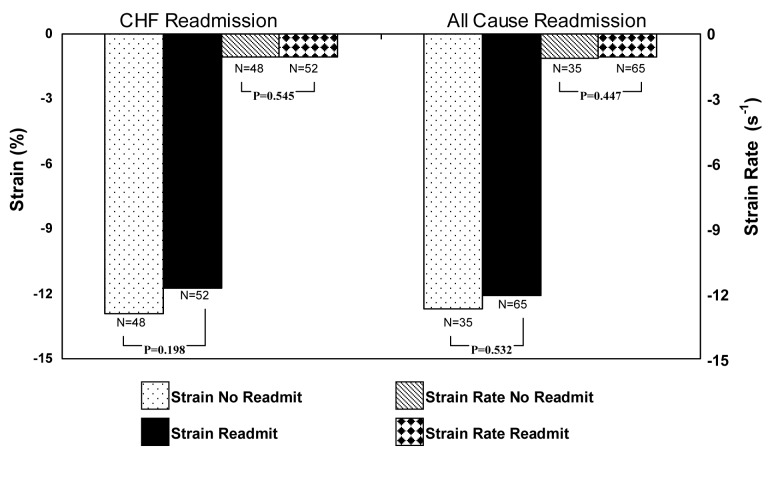
Systolic strain and strain rate values for CHF readmission and for All-Cause readmission.

Compared with controls, systolic strain was reduced in all HFNEF patients, irrespective of readmission status (-12.3% versus -15.4%, P < 0.001, Fig. 1). Systolic strain rate was not different between HFNEF patients and controls (-1.07 s^-1^ versus -1.07 s^-1^, P = 0.957) (Table 2). Systolic strain, however, did not differ significantly between patients readmitted for CHF and those who remained free from CHF readmission (-11.7% for those readmitted compared with -12.9% for those free from readmission, P = 0.190, Fig. 2), and systolic strain rates also were similar (-1.05 s^-1^ versus -1.09 s^-1^, P = 0.545). A logistic regression analysis was performed employing readmission status as the dependent variable and all recorded study variables as covariates. Only E/e’ (OR 1.135, 95% CI 1.004-1.282, P = 0.042) was found to be an independent predictor for readmission for HFNEF.

**Table 2:** Baseline Demographics and Echocardiographic Parameters for All HFNEF Patients and Controls

Variable	HFNEF (n = 100)	Controls (n = 50)	P value
Age (years)	76	60	< 0.001*
Males (n, %)	34 (0.34)	32 (0.64)	< 0.001*
Females (n, %)	66 (0.66)	18 (0.36)	< 0.001*
Hypertension (n, %)	45 (0.45)	28 (0.54)	0.204
Diabetes (n, %)	42 (0.42)	10 (0.20)	0.007*
LVEF (%)	65	64	0.189
LVIDd (cm)	4.4	4.3	0.182
LVIDs (cm)	2.9	2.7	0.095
Septal thickness (cm)	1.25	1.14	0.003*
Post wall thickness (cm)	1.19	1.09	0.003*
LA dimension (cm)	4.1	3.4	< 0.001*
Peak mitral E (cm/s)	94	69	< 0.001*
E/a	1.5	1.0	< 0.001*
e’ (cm/s)	8.0	9.0	0.069
E/e’	12.9	8.3	< 0.001*
Sa (cm/s)	7.5	9.7	< 0.001*
Systolic strain (%)	-12.3	-15.4	< 0.001*
Systolic strain rate (s^-1^)	-1.07	-1.07	0.957

^*^values are statistically significant at P < 0.05.

When analyzed for all-cause readmission (a secondary endpoint), 65 patients were readmitted while 35 patients remained free from readmission (Table 3). With the exception of diabetes, study variables were similar between the 2 groups. Diabetes was present in 34 patients (52%) in readmitted patients compared with 8 patients (23%) among those who remained free from readmission (χ^2^ = 8.10, P = 0.004). Systolic strain was abnormally low in both groups, but not significantly different between them (-12.1% versus -12.7%, P = 0.532). Strain rate also was similar for both readmitted and nonreadmitted patients (-1.05 s^-1^ versus -1.11 s-^1^, P = 0.447). In a logistic regression analysis employing readmission as the dependent variable, only diabetes emerged as an independent predictor for all-cause readmission in this population (OR 4.524, 95% CI 1.047-19.543, P = 0.043).

**Table 3:** Baseline Demographics and Echocardiographic Parameters for All-cause Readmission

variable	Not Readmitted (n=35)	Readmitted, all-cause (n=65)	P value
Age (years)	76	77	0.666
Index Length of stay (days)	5.09	5.14	0.940
Males (n, %)	8 (0.23)	26 (0.40)	0.121
Females (n, %)	27 (0.77)	39 (0.60)	0.121
Hypertension (n, %)	16 (0.46)	29 (0.45)	0.916
Diabetes (n, %)	8 (0.24)	34 (0.52)	0.004*
BNP (pg/mL)	912	925	0.788
LVEF (%)	67	65	0.069
LVIDd (cm)	4.3	4.5	0.108
LVIDs (cm)	2.8	3	0.104
Septal thickness (cm)	1.2	1.3	0.361
Post wall thickness (cm)	1.2	1.2	0.328
LA dimension (cm)	4.2	4.1	0.858
Peak mitral E (cm/s)	94	96	0.755
E/a	1.7	1.4	0.240
E’ (cm/s)	8.3	7.9	0.726
E/e’	11.2	13.8	0.104
Sa (cm/s)	7.6	7.5	0.840
Systolic strain (%)	-12.72	-12.08	0.532
Systolic strain rate (s^-1^)	-1.11	-1.05	0.447

^*^values are statistically significant at P < 0.05.

## Discussion

In a consecutive series of patients hospitalized for HFNEF, systolic strain was reduced by 20% compared with controls [18, 19]. To our knowledge, ours is the first effort to attempt to correlate systolic strain and/or strain rate with a clinical endpoint. Although neither systolic strain nor strain rate predicted increased risk for hospital readmission, the tantalizing possibility persists that these variables might provide a sensitive measure of abnormal myocardial contraction even when echocardiographically derived indices of left ventricular volume (and thus ejection fraction) are normal. In patients with hypertrophic cardiomyopathy, for example, regions that demonstrate reduced systolic strain produce delayed enhancement as measured by cardiac MRI, suggesting that myocardial fibrosis (and consequent myocardial dysfunction) are both present, despite a normal ejection fraction [20]. Reduced systolic strain in regions of increased wall thickness confirms that contractility is also decreased in regions which can be expected to manifest abnormal relaxation [21, 22]. These data support the notion that subtle abnormalities of systolic function might comprise part of the underlying pathophysiology of HFNEF.

We found that systolic strain was significantly impaired in HFNEF patients while systolic strain rate values did not differ from controls. This finding suggests that in patients with HFNEF, (and perhaps in patients mild systolic heart failure), longitudinal shortening may be impaired while the rate of myocardial deformation remains preserved. The observed decrease in systolic annular velocity (Sa) is consistent with this possibility. A differential between deformation and shortening would support the hypothesis that the endocardial and epicardial longitudinally-aligned fibers are more susceptible to hemodynamic loads as compared to the mid-myocardial, circumferentially-aligned fibers. It is also possible that intracellular calcium handling may play a role in this pathophysiology as longitudinal shortening begins in isovolumic contraction, whereas radial contraction is slightly delayed.

An alternative possibility is that reduced systolic strain and strain rate values simply reflect elevated filling pressure. While some investigators assert that abnormal strain and strain rate in HFNEF patients is a simply a function of intrinsic diastolic dysfunction [23], others have reported a correlation between reduced systolic strain and strain rate and elevated end diastolic pressure, and have concluded that such abnormalities are probably load-dependent [24]. At discharge, higher left ventricular filling pressures are an accurate predictor of repeat hospitalization and increased mortality [25, 26]. While the overall accuracy of E/e’ for prediction of diastolic filling pressure remains the subject of intense debate [27, 28], our results agree with findings from several recent studies examining the relation between elevated E/e’ at discharge and increased readmission rates [29, 30]. Thus, E/e’ also might provide a ready measure of response to treatment and a useful noninvasive marker for elevated filling pressures, especially in patients with HFNEF.

The presence of diabetes proved to be an adverse marker of outcome in our patients, as it has for numerous other populations with cardiovascular disease. Diabetes has long been a marker for multiple morbidities, including infection, vascular disease, heart failure, and most ominously, ischemic heart disease. All these conditions would no doubt contribute to all-cause readmission in our study group. By contrast, BNP levels, although elevated in our patients, were indistinguishable between readmission and non-readmission groups, probably because they were obtained early in the course of hospitalization. BNP levels have been shown to predict severity of diastolic dysfunction in HFNEF patients [31] and higher levels at discharge have also predicted increased readmission rates [32, 33].

Systolic strain and strain rate were measured only in the apical 4 chamber view, raising the possibility that a more comprehensive assessment, including apical 2 chamber and apical long axis views, might have altered our results. The difficulty of obtaining images of sufficient quality in these alternate views highlights a major limitation of any grey scale imaging technique, which is the essential requirement for high resolution of the endocardial surface-blood interface. The advantage of the Velocity Vector Imaging© system, however, is that it allows for analysis of any DICOM image, and thus does not require raw data as is necessary with other speckle tracking systems. Without such a tool, an investigation such as ours would not be possible.

In summary, we found systolic strain and strain rate to be abnormally reduced in a consecutive series of patients hospitalized for HFNEF. Our data provide further incentives for ongoing close examination of the role of strain imaging in the assessment of clinical cardiac performance.
